# 

**Published:** 1897-02

**Authors:** 


### CONTENTS—FEBRUARY, 1897.—VOL. XII., NO. 8.

ORIGINAL CONTRIBUTIONS—

A Case of Chondro-Sarcoma of the Chest. Wilson T. Davidson, M. D. . . . 415
Clinical Lecture. John V. Shoemaker, M. D. . . . . . . . . . . . . . . 422
Treatment of Abortion. Q. C. Smith, M. D . . . . . . . . . . . . . . . . 429
Rheumatism. E. B. Jackson, M. D. . . . . . . . . . . . . . . . . . . . . 434
Castration. T. J. Pugh, M. D. . . . . . . . . . . . . . . . . . . . . . . 440
Normal Labor. J. W. Cole, M. D. . . . . . . . . . . . . . . . . . . . . . 441
Gunshot Fracture of Clavicle.—Notes of Case. R. B. Longmire, M. D. . . 443

CORRESPONDENCE—

Help Wanted . . . . . . . . . . . . . . . . . . . . . . . . . . . . . . . 445
New Treatment of Carbuncle. Reply to Dr. Morris . . . . . . . . . . . . 445
Associated Physicians and Surgeons of Santa Clara Valley . . . . . . . . 447

ABSTRACTS AND SELECTIONS—

The Treatment of Warty Growths of the Genitals . . . . . . . . . . . . . 451

EDITORIAL—

Strike Now for a Medical Bill . . . . . . . . . . . . . . . . . . . . . . 453
A Warm Discussion . . . . . . . . . . . . . . . . . . . . . . . . . . . . 455
No Row . . . . . . . . . . . . . . . . . . . . . . . . . . . . . . . . . . 456
An Unfortunate Young Man . . . . . . . . . . . . . . . . . . . . . . . . 457
Nasal Catarrh . . . . . . . . . . . . . . . . . . . . . . . . . . . . . . 459

THE JOURNAL'S OWN PAGE . . . . . . . . . . . . . . . . . . . . . . . . . 452
SOCIETY NOTES . . . . . . . . . . . . . . . . . . . . . . . . . . . . 462-463
MEDICAL NEWS AND MISCELLANY . . . . . . . . . . . . . . . . . . . . 463-466
BOOK NOTICES . . . . . . . . . . . . . . . . . . . . . . . . . . . . 466-471
PUBLISHERS' NOTES . . . . . . . . . . . . . . . . . . . . . . . . . 471-474

